# Macroalbuminuria as a marker of cardiac damage in patients with type 2 diabetes: insights from multimodality cardiac imaging and biomarkers

**DOI:** 10.1007/s13105-026-01236-5

**Published:** 2026-09-21

**Authors:** Xabier Irazusta Olloquiegui, Saioa Echeverria Andueza, Marina Pascual Izco, Gonzalo Luis Alonso Salinas, Loreto Fernández Lorente, Susana Ravassa Albéniz, Sergio M. Solis-Barquero, Verónica Aramendía-Vidaurreta, José María Mora-Gutiérrez, Josune Orbe, Maria A. Fernández-Seara, Nuria García-Fernández

**Affiliations:** 1 Department of Cardiology, Clínica Universidad de Navarra, Pamplona, Spain; 2 Department of Endocrinology, Clínica Universidad de Navarra, Pamplona, Spain; 3 Instituto de Investigación Sanitaria de Navarra (IdiSNA), Pamplona, Spain; 4 Hospital Universitario de Navarra, Universidad Pública de Navarra, Pamplona, Spain; 5 Department of Nephrology, Clínica Universidad de Navarra, Pamplona, Spain; 6 Program of Cardiovascular Diseases, CIMA Universidad de Navarra, Pamplona, Spain; 7 CIBERCV, Madrid, Spain; 8 Department of Radiology, Clínica Universidad de Navarra, Pamplona, Spain; 9 Department of Hematology, Clínica Universidad de Navarra, Pamplona, Spain; 10 RICORS2040-Renal, Pamplona, España; 11 Renal MRI Network, https://renalmri.org/

**Keywords:** Chronic kidney disease, Macroalbuminuria, Cardiac magnetic resonance (MRI), Soluble ST2, Type 2 diabetes, Cardiac imaging

## Abstract

Background and aims: Albuminuria reflects renal and cardiovascular damage in type 2 diabetes (T2D), but its association is not fully understood. We evaluated the association of macroalbuminuria with cardiac structure and function. Methods: Cross-sectional study of 49 adults with T2D and stage 3 chronic kidney disease (CKD), stratified by macroalbuminuria, and 20 controls. Cardiac assessment included cardiac magnetic resonance (MRI), transthoracic echocardiography (TTE) and nine serum biomarkers, including soluble suppression of tumorigenicity 2 (sST2), a biomarker of myocardial stress and fibrosis. Results: Patients with T2D exhibited higher circulating levels of TIMP-1, MMP-10, neutrophil gelatinase-associated lipocalin (NGAL), NT-proBNP and hs-TnT than controls (*p* < 0.05). Among patients with T2D, macroalbuminuria was associated with an additional increase in TIMP-1 (*p* < 0.05). Patients with T2D had higher T1 values in MRI scans, particularly those with macroalbuminuria (1051 ms [IQR: 1035–1074] vs. 1009 ms [IQR: 989–1020] in controls, *p* < 0.001). Although sST2 levels were higher in patients with T2D than in controls and tended to be higher in those with macroalbuminuria, these differences did not reach statistical significance. Nevertheless, higher T1 values were associated with higher sST2 levels, particularly in patients with macroalbuminuria (p for interaction < 0.01). Among patients with macroalbuminuria, high sST2 levels were associated with higher T1 values (1071 ms [IQR: 1052–1087] vs. 1029 ms [IQR: 1013–1043], *p* < 0.05). Conclusions: In T2D and stage 3 CKD, macroalbuminuria identifies a subgroup with more cardiac structural and functional abnormalities, particularly with elevated sST2. Prospective validation is needed.

**Supplementary Information:**

The online version contains supplementary material available at https://doi.org/10.1007/s13105-026-01236-5.

## Key Points

Macroalbuminuria in T2D identifies a high-risk cardiac phenotype despite similar eGFR.

Elevated sST2 is linked to albuminuria and cardiac fibrosis and inflammation.

High sST2 and macroalbuminuria are linked to higher T1 and impaired myocardial strain.

These findings support an inflammatory-fibrotic cardio-renal phenotype in disease.

**Supplementary Information:**

The online version contains supplementary material available at https://doi.org/10.1007/s13105-026-01236-5.

## Introduction

The close relationship between type 2 diabetes (T2D), chronic kidney disease (CKD), and heart failure (HF) is well recognized and has reached pandemic proportions. Beyond the growing understanding of the pathophysiological mechanisms linking these conditions, evidence from large, population-based registries further supports their close clinical interconnection. In a British cohort of patients with a new diagnosis of HF, hospitalisation and mortality rates were higher among individuals with T2D or CKD, and were highest in those with both comorbidities [1]. Similarly, the Spanish CARDIOREN registry, which studied 1,107 ambulatory patients with chronic HF, found that almost 70% had concomitant kidney disease, as defined by a reduced estimated glomerular filtration rate (eGFR) and/or albuminuria [2].

Diabetes is the leading cause of CKD, with approximately 40% of patients with T2D developing CKD, a condition affecting 10–15% of the global population [3]. Diabetes is also a leading cause of HF, both with and without systolic dysfunction [4]. This association was classically attributed to the higher incidence of coronary heart disease in patients with diabetes. However, compelling evidence supports the direct effects of diabetes on left ventricular function and remodelling [5]. Structural myocardial alterations, including increased interstitial fibrosis, myocyte apoptosis, and extracellular matrix expansion, have been identified in patients with diabetes and in animal models, supporting the concept of diabetic cardiomyopathy [5, 6].

A recent statement from the American Heart Association (AHA) on cardiovascular-kidney-metabolic (CKM) syndrome highlights the complex relationships among T2D, CKD, and cardiovascular disease. This framework underscores the relevance of metabolic risk factors and inflammatory and fibrotic pathways in both renal and cardiac disease [7].

In addition to being an established biomarker for diagnosing and tracking the progression of CKD, albuminuria is recognised as a marker of cardiovascular risk. In T2D patients, it independently predicts cardiovascular disease, and a higher urinary albumin-to-creatinine ratio (UACR) is associated with an increased risk of major adverse cardiovascular events, even below the conventional threshold for abnormality. Furthermore, albuminuria may reflect systemic vascular and endothelial injury affecting multiple organs [8, 9].

T2D creates an environment in which both the kidney and the heart may be more susceptible to inflammatory and fibrotic injury. The early detection and management of cardiorenal involvement in these patients remains challenging due to the invasive nature of tissue biopsies, and routine clinical measurements may not detect subclinical disease. With regard to cardiac involvement, non-invasive imaging modalities, particularly advanced transthoracic echocardiography (TTE) and cardiac magnetic resonance imaging (MRI), are promising tools.

Conventional echocardiography enables assessment of left ventricular dimensions (mass, diameters, and volumes) and systolic and diastolic function. The most commonly used parameter of left ventricular systolic function is the left ventricular ejection fraction (LVEF), with values ≥ 50% generally considered within the normal range. Nevertheless, a substantial proportion of patients with HF and T2D have LVEF values within the normal range. Consequently, LVEF may be insufficient to identify diabetic patients at higher risk of subclinical cardiac impairment. Speckle-tracking assessment of left ventricular longitudinal myocardial deformation (strain) enables detection of subclinical left systolic dysfunction. Up to 45% of patients with diabetes may have impaired left ventricular global longitudinal strain (LVGLS) despite preserved LVEF [10]. Unlike LVEF, LVGLS may also identify subclinical cardiac damage in early-stage CKD [11].

Additionally, MRI can characterize myocardial structure and function at global and regional levels, providing information on fibrosis (through T1 mapping), inflammation, oedema (through T2 mapping), and microvascular integrity (through perfusion imaging). MRI therefore allows non-invasive myocardial tissue characterization, and may detect early abnormalities in diabetic patients, even in the absence of overt cardiac disease [12].

Circulating biomarkers may provide complementary information on inflammatory and profibrotic pathways involved in renal and cardiac injury. The roles of matrix metalloproteinases (MMPs) and their inhibitors (TIMPs) in diabetic kidney disease (DKD) have been described [13]. Other potential biomarkers include lipocalin-2 (NGAL), a marker of tubular injury and inflammation, and soluble interleukin-33 receptor-related protein (sST2), which may attenuate cardioprotective interleukin-33 signalling. It should be noted that higher circulating sST2 concentrations are associated with myocardial stress, inflammation, fibrosis, and adverse outcomes in HF [14–21].

This context has important clinical implications, as therapies that reduce albuminuria, including angiotensin-converting enzyme inhibitors, angiotensin receptor blockers, and sodium-glucose cotransporter-2 (SGLT2) inhibitors, have consistently been shown to improve both cardiovascular and renal outcomes in patients with diabetes, CKD, and HF [22]. In patients with HF, these benefits extend across the spectrum of LVEF, including significant reductions in HF hospitalizations [23–28]. Therefore, identifying early markers of cardiorenal involvement may improve risk stratification and facilitate timely implementation of disease-modifying therapies.

We hypothesised that among patients with T2D and stage 3-CKD, macroalbuminuria would be associated with a more adverse cardiac phenotype characterised by greater structural and functional abnormalities on MRI and advanced TTE. In addition, these associations would be linked to circulating biomarkers of inflammatory and fibrotic cardiorenal pathways.

## Materials and methods

### Study population

Subjects were enrolled in the study between July 2021 and July 2023. Adults with T2D and a clinical diagnosis of CKD were eligible if their estimated eGFR was 30–59 mL/min/1.73 m2 (KDIGO stage 3 CKD), regardless of albuminuria status. A total of 49 patients (34 men [69.4%] and 15 women [30.6%]) aged 18–79 years, were included. Key exclusion criteria were current corticosteroids or immunosuppressive treatment, ongoing inflammatory, autoimmune, or neoplastic processes, and/or suspicion of nephropathy not related to diabetes. Additionally, 20 healthy volunteers with no history of T2D or renal disease and no chronic medication were prospectively enrolled. The study protocol was approved by the Committee of Ethics for Research at the University of Navarra approval nº 2021.008 Mod1), and all participants provided written informed consent.

### **Data and measurements**

At baseline, the following were recorded: age; sex; body mass index (BMI); heart rate; diagnosis of hypertension; medication use; and major comorbidities. Blood sampling, TTE and cardiac MRI were all performed on the same day.

### Renal function data

eGFR was estimated using the 2021 CKD Epidemiology Collaboration CKD-EPI Creatinine Eq. (2021) [29]. UACR was measured in a spot urine sample and expressed as milligrams of albumin per gram of creatinine. Patients with T2D were categorised as having macroalbuminuria (UACR ≥ 300 mg/g) or microalbuminuria (UACR < 300 mg/g).

### Echocardiography and serum biomarkers

All participants underwent TTE using a Vivid™ E95 4D system equipped with an M5S 3.5-MHz transducer (GE Healthcare, USA). Standard two-dimensional images were acquired and stored in digital cineloop format for offline analysis. TTE assessment included left ventricular (LV) mass index, ejection fraction, diastolic function, and myocardial strain parameters, as detailed in the Supplementary Methods. In addition, circulating biomarkers of myocardial fibrosis, extracellular matrix remodeling, cardiac stress, and kidney injury were measured in serum samples using commercially available assays. PICP, sST2, MMP-10, TIMP-1 and NGAL (lipocalin-2) were measured using ELISA; CICP using EIA; and MMP-1 and TIMP-1 using AlphaLISA assays. Full commercial information is included in the Supplementary Methods. NT-proBNP and high-sensitivity troponin T (hsTnT) concentrations were determined by electrochemiluminescence immunoassay as previously described.

#### **Cardiac MRI protocol**

Cardiac MRI was performed using a 1.5 Tesla scanner with advanced sequences including cine, T1, T2, T2* mapping, and arterial spin labeling (ASL) for perfusion imaging. Detailed imaging protocols, including specific sequences, parameters, and methods for myocardial blood flow calculation, are available in the supplementary data.

#### Statistical analyses

Sample size was estimated to detect a 17 ms difference in native myocardial T1 values between T2D patients with and without macroalbuminuria, assuming a standard deviation of 20 ms (effect size of Cohen’s d = 0.85), according to previous pilot data. With 80% power and a two-sided alpha of 0.05, 46 patients were required for an independent-samples Student’s t test (G*Power 3.1.9.7). Therefore, the available sample size was considered sufficient for the planned analysis.

Continuous variables are expressed as median with 25th and 75th percentiles, and categorical variables as numbers and percentages. Clinical characteristics were compared across groups using the χ2 test for categorical variables, and the Kruskal Wallis test followed by Mann-Whitney U-test for continuous variables. Trends across ordered groups were assessed using Spearman rank correlation for continuous variables and the Cochran-Armitage trend test for categorical variables.

Non-normally distributed variables were logarithmically transformed prior to analysis. Interaction analyses were performed to assess whether UACR, modelled continuously or categorically as macroalbuminuria (UACR ≥ 300 mg/g), modified the associations between circulating biomarkers and MRI parameters. Linear regression models included the biomarker as the dependent variable, the MRI parameter, the UACR variable, and their interaction term as independent variables. Significant interaction p-values were confirmed after correction for multiple testing using the Benjamini-Hochberg procedure, with a false discovery rate of 5%.

Significant models were further adjusted for age, sex, and systolic blood pressure (SBP). Differences in MRI, echocardiographic parameters, and circulating cardiomyocyte biomarkers among T2D patients stratified by macroalbuminuria status and sST2 levels (below vs. above the median of 43.9 ng/mL) were analyzed using analysis of covariance, adjusted for the same covariates.

Heteroscedasticity and residual normality were assessed graphically using scatter plots and Q-Q plots, respectively. If outlying residual observations were found, analyses were confirmed by robust regression (R package robustbase). Multicollinearity was excluded if variance inflation factor values were lower than 5.

All tests were two-sided, with statistical significance set at *P* ≤ 0.05. Analyses were performed using R software (version 4.4.1).

## Results

### Demographics

The baseline characteristics of the 49 patients with T2D and 20 healthy participants are summarized in Table 1. In addition to other tendencies shown in this table, systolic and diastolic blood pressure and urea levels increased, whereas eGFR and haemoglobin concentrations decreased, across the ordered groups (p for trend ≤ 0.03).

**Table 1:** Clinical characteristics of controls and T2D patients categorized according to the presence or absence of macroalbuminuria

	Controls (*n* = 20)	T2D patients			*P* for trend
		Without macroalbuminuria (*n* = 35)		With macroalbuminuria (*n* = 14)	
Age, years	66.0 (64.0 to 69.0)	76.0 (68.5 to 79.5)*		70.5 (68.2 to 75.8)	0.021
Men, n(%)	13 (65)	22 (62.9)		12 (85.7)	0.252
BMI, kg/m2	23.6 (22.0 to 26.8)	29.7 (26.5 to 32.2)*		28.3 (26.5 to 33.9)*	0.003
BP					
Systolic, mmHg	140 (130 to 145)	146 (131 to 154)		167 (139 to 180)*†	0.003
Diastolic, mmHg	83.0 (70.0 to 88.0)	79.0 (70.0 to 86.5)		91.0 (83.5 to 95.8)*†	0.008
Laboratory results					
HbA1c (%)	5.4 (5.3 to 5.7)	7.1 (6.6 to 7.6)*		6.8 (6.1 to 7.6)*	0.115
Total cholesterol, mg/dl	216 (190 to 236)	170 (139 to 192)*		148 (123 to 164)*†	< 0.001
HDL, mg/dl	56.5 (50.5 to 68.0)	47.0 (34.0 to 57.0)*		41.0 (36.2 to 49.8)*	0.001
LDL, mg/dl	136 (115 to 151)	88.0 (66.0 to 116)*		58.5 (53.2 to 84.5)*†	< 0.001
UACR	10.0 (5.0 to 11.1)	33.0 (18.5 to 113)*		627 (548 to 1458)*†	< 0.001
Urea, mg/dL	42.0 (40.0 to 45.0)	61.0 (45.0 to 70.2)*		75.0 (61.8 to 82.8)*†	< 0.001
Hb, mg/dl	14.9 (13.8 to 15.6)	13.8 (12.4 to 15.1)*		13.4 (13.0 to 14.3)*	0.028
Creatinine, mg/dL	0.9 (0.8 to 1.0)	1.4 (1.3 to 1.6)*		1.7 (1.3 to 2.0)*	< 0.001
CKDEPI creatinine, ml/min/1.73 m2	86.0 (78.5 to 90.6)	41.0 (35.5 to 47.0)*		36.0 (32.2 to 55.5)*	< 0.001
Comorbidities, n(%)					
Hypertension	4 (20)		33 (94.3)*	14 (100)*	< 0.001
Stroke	0 (0.0)		4 (11.4)	1 (7.1)	
Diabetic retinopathy	0 (0.0)		7 (20.0)	6 (42.9)	
IDCM	0 (0.0)		2 (5.7)	1 (7.1)	
Valvular disease	0 (0.0)		2 (5.7)	0 (0.0)	
AF	0 (0.0)		2 (5.7)	0 (0.0)	
Medications, n(%)					
Oral antidiabetic (any)	0 (0.0)		25 (71.4)	11 (78.6)	
Including iSGLT2	0 (0.0)		9 (25.7)	6 (42.9)	
Oral antidiabetic + Insulin	0 (0.0)		12 (34.3)	8 (57.1)	
B-blocker	0 (0.0)		12 (34.3)	6 (42.9)	
ACE inhibitor/ARB	3 (15)		28 (80)*	13 (92.9)*	< 0.001
Statins	4 (20)		29 (82.9)*	12 (85.7)*	0.807
ASA/P2Y12 inhibitor	0 (0.0)		13 (37.1)	10 (71.4)†	

Continuous data are presented as median (interquartile range). T2D: Type II diabetes mellitus; BMI: Body Mass Index; BP: Blood Pressure; HbA1C: Hemoglobin A1c; HDL: High Density Lipoprotein; LDL: Low Density Lipoprotein; UACR: Urine Albumin-Creatinine Ratio; CKD-EPI: Chronic Kidney Disease Epidemiology Collaboration; AF: Atrial Fibrillation; B-blocker: Beta blocker; ACE inhibitor/ARB: Angiotensin Converting Enzyme Inhibitors/ Angiotensin Receptor Blockers; ASA: Acetylsalicylic acid. *P < 0.05 versus Controls; †*P* < 0.05 versus T2D without macroalbuminuria

### Cardiac imaging parameters

TTE and MRI parameters are summarized in Table 2. Compared with controls, patients with T2D had increased left ventricular mass, impaired atrial reservoir strain and worse LVGLS values. These abnormalities were more pronounced in patients with macroalbuminuria.

**Table 2:** Cardiac imaging parameters (both echocardiography and MRI) in controls and T2D patients categorized according to the presence or absence of macroalbuminuria

	Controls (*n* = 20)	T2D patients		*P* for trend
		Without macroalbuminuria (*n* = 35)	With macroalbuminuria (*n* = 14)	
Echocardiography				
Left ventricle				
LVMI, g/m2	76.6 (66.6 to 90.2)	85.0 (66.9 to 112.8)	94.6 (83.6 to 123.7)*	0.019
LVEDVi, mL/m2	45.8 (38.8 to 51.4)	32.6 (28.7 to 42.8)	47.2 (31.4 to 57.0)†	0.491
LVESVi, mL/m2	14.3 (12.7 to 16.2)	9.8 (7.0 to 12.3)	16.5 (8.7 to 24.1)†	0.644
LVEF	65.3 (63.1 to 68.9)	73.1 (68.2 to 76.3)	66.2 (58.8 to 74.4)	0.426
E/A ratio	1.0 (0.8 to 1.1)	0.7 (0.6 to 0.9)*	0.7 (0.6 to 0.8)*	0.008
E/e’	8.5 (6.5 to 9.9)	11.1 (8.2 to 13.9)*	10.0 (9.5 to 11.5)	0.030
GLS, %	-19.5 (-22.3 to -17.9)	-17.2 (-20.0 to -15.0)*	-15.9 (-17.7 to -13.9)*	0.001
Right ventricle				
GLS, %	-20.3 (-24.6 to -18.2)	-20.4 (-22.4 to -16.1)	-19.6 (-22.6 to -18.3)	0.685
FWLS, %	-25.0 (-29.9 to -22.0)	-25.1 (-28.4 to -19.8)	-25.8 (-28.6 to -22.1)	0.591
Atria				
LAD, mm	33.0 (31.2 to 39.0)	38.0 (34.0 to 45.0)*	39.0 (35.0 to 42.0)*	0.031
LAVi, mLm2	29.1 (19.2 to 31.4)	23.0 (17.3 to 31.3)	21.7 (17.1 to 35.3)	0.510
LA Strain cond.	-17.2 (-25.0 to -13.6)	-12.3 (-16.7 to -8.3)*	-9.4 (-18.8 to -5.9)*	0.001
LA Strain pump	-15.5 (-19.3 to -14.2)	-11.1 (-18.9 to -5.9)	-16.2 (-20.9 to -13.8)	0.720
LA Strain reserve.	33.8 (29.3 to 39.7)	27.4 (19.4 to 34.7)*	26.4 (22.0 to 36.6)*	0.017
MRI				
MBF with ASL	1.3 (1.1 to 2.0)	1.1 (0.8 to 1.5)*	1.2 (0.9 to 1.5)	0.097
Myocardial T1, ms	1009 (989 to 1020)	1038 (1009 to 1057)*	1051 (1035 to 1074)*	< 0.001
Myocardial T2, ms	47.8 (47.1 to 49.7)	48.8 (46.7 to 50.5)	48.6 (48.1 to 49.9)	0.255
Myocardial T2*, ms	32.9 (31.3 to 34.5)	32.0 (30.0 to 37.0)	34.2 (31.1 to 38.4)	0.485

Data are presented as median (interquartile range). T2D: Type II diabetes mellitus; LVMI: Left Ventricle Mass index; LVEDVi: Left Ventricle End Diastolic Volume index; LVESVi: Left Ventricle End Systolic Volume index; LVEF: Left Ventricle Ejection Fraction; FWLS: Free wall longitudinal strain; GLS: Global Longitudinal Strain; LAD: Left Atrial Diameter; LAVi: Left Atrial Volume index; LA Strain cond.: Left Atrial Strain conduit; LA Strain pump: Left Atrial Strain pump; LA Strain reserve: Left Atrial Strain reservoir; MRI: Cardiac Magnetic Resonance Imaging; MBF: Myocardial Blood Flow; ASL: Arterial Spin Labeling. *P < 0.05 versus Controls; †*P* < 0.05 versus DM without macroalbuminuria

Both T2D groups, exhibited higher native myocardial T1 values compared to controls (*p* < 0.05 for each comparison). Furthermore, T2D patients with macroalbuminuria showed slightly higher myocardial T1 levels compared to those without macroalbuminuria, although this difference was not statistically significant (Table 2).

### Biomarkers

Serum concentrations of PICP, CITP, MMP-1, TIMP-1, MMP-10, NGAL, sST2, NT-proBNP, and hs-TnT, as well as the CITP: MMP-1 ratio, are summarised in Supplementary Table S1. Compared with the control group, patients with T2D exhibited significantly higher levels of the extracellular matrix remodelling biomarker TIMP-1, MMP-10, NGAL and NTproBNP and hs-TnT (*p* < 0.05). However, no significant differences were observed for PICP, CITP, MMP-1, sST2 or the corresponding collagen turnover ratios. Among patients with T2D, those with macroalbuminuria exhibited further increases in TIMP-1 concentrations (*p* < 0.05).

### Analyses of interaction

**Fig. 1:**
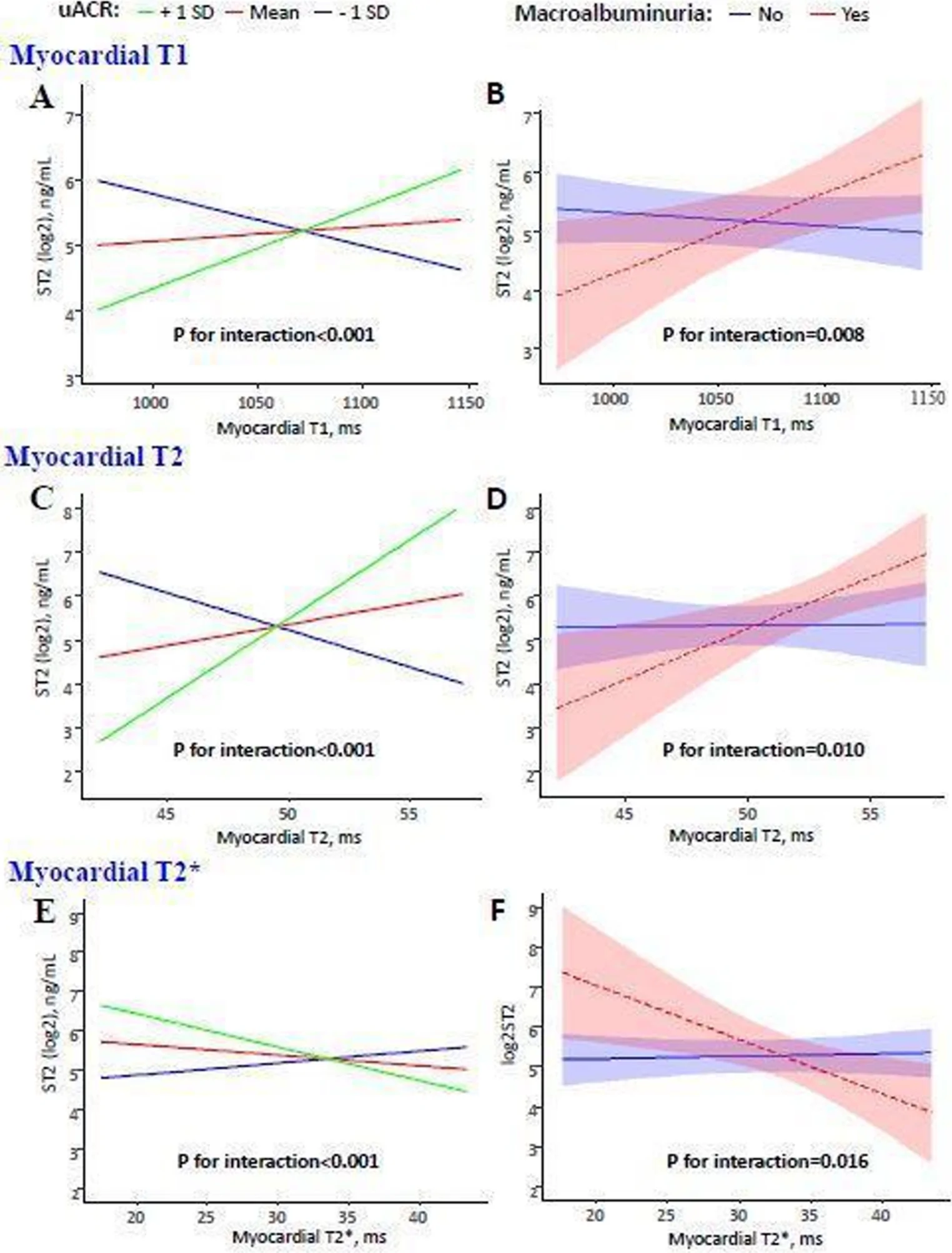
Influence of macroalbuminuria on the associations between sST2 and MRI parameters in T2D patients. Regression lines show log2-transformed sST2 values as a function of myocardial T1 (**A**, **B**), T2 (**C**, **D**) and T2* (**E**, **F**) MRI parameters from linear regression analyses including an interaction term between each MRI parameter and the urine albumin/creatinine ratio (UACR) or the presence or absence of macroalbuminuria (UACR > 300 mg/g), adjusted for age, sex, and systolic blood pressure. Interaction analyses with UACR (**A**, **C**, **E**) are presented as 1 standard deviation (SD) below (blue line) and above (green line) the UACR mean (red line), and interaction analyses with macroalbuminuria (**B**, **D**, **F**) are presented as lines and color areas showing the 95% confidence intervals for patients without (blue) or with (red) this alteration. UACR: Urine Albumin-Creatinine Ratio; sST2: Soluble suppressor of tumorigenicity-2

Macroalbuminuria significantly modified the associations of serum sST2 with MRI parameters (T1, T2, and T2*) (adjusted p for interaction = 0.008, 0.010 and 0.016, respectively) (Fig. 1). Higher sST2 levels were associated with higher T1 and T2 values and lower T2* values among patients with macroalbuminuria, whereas no corresponding associations were observed among those without macroalbuminuria (Fig. 1). No significant interactions were identified for the other evaluated biomarkers (Supplementary Table S2).

To aid interpretation of these interactions, we conducted a post hoc analysis to examine whether patients with both macroalbuminuria and elevated sST2 levels had more advanced cardiac remodelling or dysfunction.

Patients with macroalbuminuria and elevated sST2 levels exhibited higher native T1 and T2 values (Table 3) and less negative LVGLS than patients with low sST2 concentrations, regardless of macroalbuminuria status.

**Table 3:** Cardiac imaging parameters and cardiomyocyte biomarkers in T2D patients categorized according to the presence or absence of macroalbuminuria and high sST2

	No macro-albuminuria		Macro-albuminuria	
	Low sST2 (*n* = 17)	High sST2 (*n* = 18)	Low sST2 (*n* = 6)	High sST2 (*n* = 8)
MRI				
Myocardial T1, ms	1045 (1024 to 1059)	1033 (1004 to 1055)	1029 (1013 to 1043)	1071 (1052 to 1087)*^†^
Myocardial T2, ms	48.9 (46.7 to 51.0)	48.8 (46.8 to 50.3)	48.0 (47.8 to 48.1)	49.7 (49.0 to 51.4)*^†^
Myocardial T2*, ms	30.5 (27.5 to 36.8)	34.9 (30.9 to 37.5)	36.9 (34.2 to 40.6)	33.0 (29.6 to 34.4)
MBF with ASL	0.9 (0.6 to 1.4)	1.2 (0.8 to 1.7)*	1.2 (0.9 to 1.3)	1.2 (0.8 to 1.5)
Left ventricle				
LVMI, g/m^2^	82.2 (61.3 to 98.3)	88.9 (75.3 to 116)	87.1 (83.6 to 93.8)	114 (90.2 to 133)*
LVEDD, mm	44.0 (39.5 to 49.5)	46.5 (39.5 to 51.0)	45.0 (42.8 to 47.2)	46.0 (42.0 to 50.8)
LVEDVi, mL/m^2^	32.7 (28.6 to 38.5)	32.5 (30.9 to 44.7)	40.5 (32.1 to 48.3)	53.6 (32.3 to 58.5)^†^
LVESVi, mL/m^2^	9.8 (8.0 to 12.4)	9.8 (6.8 to 12.1)	12.7 (8.6 to 17.8)	20.5 (9.0 to 25.4)^†^
LVEF, %	72.4 (65.4 to 76.7)	73.4 (72.6 to 76.0)	73.0 (63.6 to 76.5)	62.8 (57.3 to 70.0)^†^
E/A ratio	0.7 (0.6 to 0.8)	0.7 (0.6 to 0.9)	0.7 (0.6 to 0.8)	0.7 (0.5 to 0.8)
DT, ms	240 (170 to 260)	220 (170 to 278)	230 (200 to 267)	200 (155 to 233)^†^
Mean Ee’	11.1 (8.4 to 14.6)	10.2 (8.5 to 13.1)	10.0 (9.8 to 10.6)	10.8 (9.1 to 11.6)
GLS, %	-17.9 (-21.1 to -16.8)	-16.5 (-18.4 to -14.5)	-18.0 (-18.4 to -16.8)	-14.1 (-15.9 to -12.9)*^†^
Right ventricle				
TAPSE, mm	22.0 (21.0 to 26.0)	21.6 (18.9 to 23.5)	26.5 (23.1 to 27.0)	22.2 (18.6 to 25.4)
GLS, %	-20.2 (-22.0 to -14.8)	-20.6 (-22.8 to -18.9)	-21.6 (-23.6 to -19.3)	-18.8 (-21.6 to -16.4)
FWLS, %	-25.0 (-27.8 to -17.8)	-26.3 (-29.0 to -22.0)	-25.6 (-28.9 to -23.6)	-25.9 (-28.4 to -21.3)
Atria				
LAD, mm	36.0 (33.0 to 40.2)	41.0 (34.0 to 48.0)	41.0 (38.0 to 41.0)	37.5 (33.5 to 43.5)
LAVi, mLm^2^	25.4 (17.3 to 29.0)	22.8 (17.3 to 32.1)	21.7 (17.1 to 35.3)	22.4 (19.2 to 34.0)
RAV, mL	33.0 (20.4 to 36.9)	19.8 (15.2 to 39.1)	27.9 (24.3 to 31.8)	26.4 (16.1 to 34.3)
LA Strain cond.	-12.1 (-17.2 to -8.6)	-12.5 (-15.6 to -8.3)	-18.9 (-19.6 to -7.9)	-7.2 (-12.0 to -4.5)^†^
LA Strain pump	-10.0 (-19.2 to -3.4)	-14.1 (-17.9 to -7.2)	-19.4 (-24.6 to -15.6)	-15.9 (-18.1 to -13.5)
LA Strain reserve.	27.9 (16.2 to 37.8)	26.9 (20.8 to 31.0)	38.0 (34.4 to 44.3)	24.1 (21.5 to 26.4)
Biomarker				
NTproBNP, ng/L	133 (95.6 to 246)	137 (66.6 to 264)	152 (63.8 to 212)	220 (93.8 to 811)
hs-TnT, ng/L	20.6 (11.7 to 28.0)	20.2 (10.4 to 32.1)	28.3 (17.8 to 34.4)	23.1 (12.1 to 29.4)

Data are presented as medians (interquartile ranges). T2D: Type II diabetes mellitus; GLS: Global Longitudinal Strain; MRI: Cardiac Magnetic Resonance Imaging; MBF: Myocardial Blood Flow; ASL: Arterial Spin Labeling; LVMI: Left Ventricle Mass index; LVEDDi: Left Ventricle End Diastolic Diameter index; LVEDVi: Left Ventricle End Diastolic Volume index; LVESVi: Left Ventricle End Systolic Volume index; LVEF: Left Ventricle Ejection Fraction; FWLS: Free wall longitudinal strain; LAD: Left Atrial Diameter; LAVi: Left Atrial Volume index; RAV: Right Atrial Volume; LA Strain cond.: Left Atrial Strain conduit; LA Strain pump: Left Atrial Strain pump; LA Strain reserve: Left Atrial Strain reservoir; NTproBNP: N-terminal brain natriuretic peptide; hs-TnT: High Sensitive Troponin. *P < 0.05 compared to no macroalbuminuria and low ST2; †*P* < 0.05 compared to macroalbuminuria and low sST2. Significant values are in bold

## Discussion

In this cohort of patients with T2D and stage 3-CKD, the presence of macroalbuminuria identified a distinct subgroup with a more adverse cardiac phenotype, despite comparable kidney function. Specifically, patients with macroalbuminuria exhibited more pronounced myocardial tissue abnormalities, as reflected by higher native T1 values, as well as worse myocardial mechanics, as evidenced by less negative LVGLS. These alterations were most pronounced in the presence of elevated circulating sST2 levels, suggesting an interplay between albuminuria, systemic fibrotic and inflammatory signalling, and subclinical myocardial remodelling. Taken together, these findings suggest that macroalbuminuria not only correlates with more severe renal impairment but is also accompanied by differences in the relationship between sST2 and cardiac tissue characteristics, underscoring its potential as a marker of increased cardiorenal vulnerability.

To the best of our knowledge, this is among the first studies to examine the association of macroalbuminuria with cardiac remodelling and function in patients with T2D in stage 3 CKD. In T2D patients with stage 3 CKD, the presence of macroalbuminuria was associated with subclinical left ventricular dysfunction, as reflected by less negative LVGLS values despite preserved LVEF. This finding is consistent with previous TTE studies showing a graded association between increasing albuminuria and impaired cardiac function in T2D. In a cohort of 915 patients, Jørgensen et al. [30] observed progressive deterioration of diastolic function across albuminuria categories, whereas impaired LVGLS was specifically associated with macroalbuminuria. Similar associations between albuminuria or diabetic nephropathy and reduced LVGLS have been reported in other studies [31, 32]. However, unlike the present study, these investigations did not specifically compare patients with and without macroalbuminuria within a population restricted to stage 3 CKD. Native T1 is sensitive to diffuse myocardial tissue abnormalities, including interstitial expansion, fibrosis and edema, but is not specific to any single process. Our MRI findings are consistent with previous reports in patients with diabetes and albuminuria, although those studies did not specifically compare patients within the same CKD stage [33]. Swoboda et al. [13] reported that microalbuminuria was associated with increased extracellular volume fraction in patients with diabetes, supporting an association between albuminuria and subclinical myocardial tissue abnormalities.

The circulating biomarker analyses provided complementary evidence of an inflammatory-fibrotic phenotype. Macroalbuminuria modified the association between sST2 and MRI tissue parameters, with higher sST2 concentrations associated with higher native T1 and T2, and lower T2* values. sST2 is an established biomarker of myocardial stress and fibrotic-inflammatory activation and is elevated in patients with both kidney disease and HF, particularly in those with diabetes [34]. As a decoy receptor for IL-33, sST2 may attenuate protective signalling, and has been associated with fibrosis, cardiac dysfunction and hypertrophy [35]. Our findings are consistent with the CKM framework proposed by the American Heart Association, which emphasizes the interdependence of metabolic dysfunction, kidney disease and cardiovascular disease and identifies inflammation and fibrosis as shared pathophysiological mechanisms across these conditions [8]. In this context, the association between higher sST2 concentrations and adverse cardiac remodeling in patients with T2D and kidney impairment may be a biomarker reflection of inflammatory-fibrotic processes linking kidney and myocardial damage. Previous studies associating elevated sST2 with myocardial fibrosis, adverse cardiac remodelling and cardiovascular outcomes provide additional support for this interpretation [36–38], although the association between sST2 and native T1 has been inconsistent [39]. Differences in study populations, imaging protocols, and kidney disease severity may partly explain this discrepancy. Our results suggest that albuminuria status may be an important effect modifier when evaluating sST2-imaging associations; however, this interpretation requires confirmation in larger prospective cohorts.

High sST2 concentrations were also associated with TTE abnormalities. In the subgroup of patients with macroalbuminuria, high sST2 was accompanied by less negative LVGLS, larger ventricular volumes and lower LVEF and deceleration time. Previous studies have linked sST2 to LV hypertrophy, diastolic dysfunction and adverse remodeling [40, 41]. Our findings extend these observations by suggesting that macroalbuminuria may identify a clinical context in which sST2 is more closely associated with subclinical cardiac structural and functional abnormalities.

These findings may also be relevant to HF with preserved ejection fraction (HFpEF), particularly in patients with T2D and CKD. Albuminuria has been independently associated with adverse cardiac remodelling, biventricular dysfunction, and worse outcomes in HFpEF [41]. In our cohort, macroalbuminuria was associated with a more adverse cardiac phenotype, but the cross-sectional design cannot determine whether albuminuria contributes directly to myocardial injury or reflects shared vascular, inflammatory and fibrotic processes. SGLT2 inhibitors and finerenone reduce HF events in patients with mildly reduced or preserved LVEF and also have kidney-protective and albuminuria-lowering effects [23–28, 42, 43]. Whether reductions in albuminuria mediate improvements in myocardial inflammation or fibrosis remains uncertain. Macroalbuminuria should therefore be considered as an accessible risk marker and a potential treatment target that warrants prospective mechanistic evaluation in cardiorenal patients with HFpEF.

From a clinical perspective, these findings suggest that macroalbuminuria may improve cardiovascular risk stratification in patients with T2D by identifying individuals with greater myocardial involvement despite similar kidney function. Although cardiac MRI is highly effective in detecting subclinical myocardial abnormalities, its routine use may be limited by cost and availability. In contrast, UACR is inexpensive, widely available, and is already part of standard diabetes care. Although our cross-sectional data cannot prove causation, the observed link between macroalbuminuria and an adverse cardiac phenotype suggests that albuminuria could help identify patients with myocardial structural abnormalities, who could potentially benefit from advanced cardiac imaging. Therefore, future prospective studies should evaluate whether using cardiac MRI in patients with albuminuria improves risk stratification and clinical decision-making.

### Study limitations

Several limitations should be taken into account when interpreting these findings. The sample size was relatively small, so the results should be regarded as preliminary and hypothesis-generating. Additionally, the cross-sectional design enables the evaluation of correlations but precludes definitive conclusions about causation. Residual confounding factors, such as variations in blood pressure and the use of cardiorenal therapies like SGLT2 inhibitors, could have impacted the observed correlations. Although extracellular volume fraction may provide a more specific estimate of interstitial expansion, we used non-contrast native T1 and T2 mapping to avoid exposure to gadolinium-based contrast agents in a population with renal impairment. These techniques are sensitive to both fibrotic and inflammatory tissue changes, but are not specific to either process. Finally, the post-hoc analyses based on the median sST2 concentration require confirmation in larger, independent cohorts.

### Conclusions

Among patients with T2D and stage 3 CKD, macroalbuminuria emerged as a key marker of a more adverse cardiac phenotype, despite comparable kidney function. The presence of macroalbuminuria was associated with unfavourable cardiac imaging features and significantly modified the relationship between circulating sST2 levels and myocardial tissue characteristics. Notably, patients with both macroalbuminuria and elevated sST2 levels had the highest native T1 and T2 values, as well as the greatest impairment in LVGLS. These findings suggest that macroalbuminuria identifies a subgroup with increased cardiorenal vulnerability, supporting the potential value of combining this readily available renal marker with circulating biomarkers and advanced cardiac imaging to improve the characterisation of subclinical cardiorenal involvement. Further prospective studies are needed to confirm these observations and determine their prognostic significance.

## Supplementary Information

Below is the link to the electronic supplementary material.

Supplementary Material 1

## Data Availability

The data supporting the findings of this study will be made available upon reasonable and well-justified request to the corresponding author.
