# Supplementary material for: Macroalbuminuria as a marker of cardiac damage in patients with type 2 diabetes: insights from multimodality cardiac imaging and biomarkers
Source: J Physiol Biochem. 2026 Sep 21;82(1):96. doi: 10.1007/s13105-026-01236-5 (PMC13593709; doi:10.1007/s13105-026-01236-5)
Supplement: Supplementary file 1 [file 13105_2026_1236_MOESM1_ESM.docx]

**Supplementary data**

**Biomarkers:** Serum PICP, CITP, and MMP1 levels were measured in all patients using an enzyme immunoassay (MicroVue CICP EIA, QuidelOrtho), a radioimmunoassay (UniQ ICTP de Orion Diagnostica), and an AlphaLISA assay (PerkinElmer), respectively; and NT-proBNP and high-sensitivity troponin T hsTnT levels were determined using an immunoelectrochemiluminescence method (Roche), as previously described (30). CITP and MMP-1 were expressed in molarity and the CITP:MMP-1 ratio was calculated in each patient. TIMP-1 and MMP10 were measured using ELISA methods as previously described (31). LCN2 determination was performed in serum, using the Human Lipocalin-2/NGAL ELISA (R&D Systems;Biovendor) with inter- and intra-assay coefficients of variation of < 10% and < 8%, respectively, and a limit of detection of 0.02 ng/mL. Soluble sST2 was measured using an EIA (sST2 Presage®, Critical Diagnostics).12 For all biomarkers the inter-assay and intra-assay coefficients of variation were lower than 10%.

**Echocardiography data:** From a parasternal view, 2D measurements included left ventricle (LV) septal (IVSd) and posterior wall thickness (PWd), and end-diastolic diameter (LVDd). LV mass index was calculated as the anatomic mass divided by body surface area. From an apical perspective, 2D LV ejection fraction (LV EF) measurements were based on end-systolic/diastolic volumes using the disc biplane technique. Regarding the analysis of diastolic function, peak early (E) and late (A) diastolic transmitral flow velocity and E/A ratio, deceleration time (DT) of E wave, pulsed wave Tissue Doppler diastolic (E′) velocities at both septal and lateral mitral annulus and E/E′ ratio as an estimate of LV filling pressure were assessed. All 2D and Doppler recordings and measurements were performed according to American Guidelines (32).

To calculate LV global longitudinal strain (GLS), 2D images from the apical four-chamber, two-chamber, and three-chamber views were acquired. To calculate right ventricle (RV) GLS and free wall longitudinal strain (FWLS), a modified apical four-chamber view for the RV was acquired. Finally, left atria (LA) strain data were calculated from the apical four-chamber view. LA was traced on the best echocardiographic image at the end diastolic phase.

All strain data were quantified using semi-automated function imaging from vendor-specific offline analysis software (Tomtec-Arena TTA 2.5, Munich, Germany).

**Cardiac mpMRI protocol:** Multiparametric cardiac magnetic resonance (mpMRI) images were acquired in a 1.5 Tesla scanner (Aera, Siemens Healthcare, Erlangen, Germany) with 18-channel body and 32-channel spine coils. The cardiac mpMRI protocol consisted in the acquisition of routine localizers, followed by anatomical and functional images using cine, myocardium T1, T2 and T2* mapping sequences, and finally, an arterial spin labelling (ASL) perfusion sequence. Relaxation parameter mapping sequences covered three short axis slices (basal, mid-ventricular, and apical) and were run in breath-holds. A cardiac flow-sensitive alternating inversion recovery (FAIR) pulsed ASL (PASL) technique was used for non-contrast perfusion quantification. Images for FAIR PASL were only acquired in the mid-ventricular short axis slice of the myocardium. Total acquisition time was approximately thirty minutes.

For T1 mapping, a high-resolution Modified Look-Locker Inversion Recovery (MOLLI) sequence (33) with a 5(3)3(0) scheme was used.14 Other imaging parameters were: matrix size = 256 x 161, field-of-view (FOV)= 350 x 333 mm2, slice thickness = 8 mm, repetition time (TR)=362.11 ms, echo-time (TE)=1.14 ms, flip angle = 35°.

For T2 mapping, a True Fast Imaging with Steady-State Free Precession (TrueFISP) with 0 ms, 25 ms and 55 ms adiabatic T2 preparation was employed (34). Other imaging parameters were: matrix size = 192 x 141, FOV = 332 x 239 mm2, slice thickness = 8 mm, TR = 234.4 ms, TE=1.09 ms, flip angle = 70°.

The T2* mapping sequence was based on a multi gradient echo (GRE) sequence with eight equally spaced echoes from 2.08 ms to 16.22 ms. Other imaging parameters were: matrix size =256 x 130, FOV = 337 x 400 mm2, slice thickness = 8 mm, TR= 772 ms, TE=2.08, 4.1, 6.12, 8.14, 10.16, 12.18, 14.2, 16.22 ms, flip angle = 20°.

Relaxation parameter maps were generated by the scanner software. Manually drawn regions-of-interest (ROIs) were used to obtain native T1, T2 and T2* values from the entire visible myocardium in the analysed mid-ventricular short axis slice.

The FAIR PASL sequence was optimized in a previous study (35). Images were acquired during diastole, using ECG gating. A fixed inversion time of 1000 ms was used. Parameters for the balanced steady-state free-precession (bSSFP) readout were: FOV=343x343 mm2, matrix=128x128, slice thickness=10 mm, isotropic pixel size=2.68x2.68 mm2, TR=5000 ms, TE=1.19 ms, flip-angle=70°. A total of 35 pairs of slice-selective and non-selective images were acquired in free breathing. Also, a baseline M0 (without pre-saturation or saturation pulses) was acquired before, during breath hold, for perfusion quantification purposes. Myocardial blood flow (MBF) was calculated for the complete myocardium, as stated in (36) after pre-processing including registration for the reduction of motion effects (37).

**Supplementary material**

**Supplementary Table S1: Biomarkers in controls and T2D patients categorized according to the presence or absence of macroalbuminria**

|  | **Controls**  **(n=20)** | **T2D patients** | | **P for trend** |
| --- | --- | --- | --- | --- |
|  |  | **Without macroalbuminuria**  **(n=35)** | **With macroalbuminuria**  **(n=14)** |  |
| **Biomarkers** |  |  |  |  |
| PICP, ng/mL | 102 (90.9 to 135) | 118 (90.8 to 158.0) | 127 (96.8 to 152) | 0.205 |
| CITP, ng/mL | 3.1 (2.5 to 5.6) | 6.0 (3.2 to 7.9) | 4.8 (2.8 to 7.3) | 0.202 |
| PICP/CITP ratio | 29.4 (17.6 to 47.7) | 23.5 (12.9 to 38.4) | 21.1 (17.9 to 41.5) | 0.384 |
| MMP1, ng/mL | 13.3 (10.5 to 19.5) | 21.0 (9.9 to 31.1) | 17.5 (11.2 to 21.4) | 0.351 |
| TIMP1, ng/mL | 172 (139 to 202) | 225 (198 to 247)* | 267 (222 to 339)*† | <0.001 |
| MMP1/TIMP1 ratio | 0.085 (0.056 to 0.11) | 0.091 (0.050 to 0.13) | 0.059 (0.040 to 0.084) | 0.146 |
| sST2, ng/mL | 32.6 (22.7 to 46.1) | 43.9 (32.0 to 55.9)* | 45.6 (23.3 to 61.6) | 0.135 |
| Lipocaline, ng/mL | 45.9 (38.4 to 54.0) | 69.1 (48.6 to 102.0)* | 76.2 (68.7 to 102.0)* | <0.001 |
| MMP10, pg/mL | 524 (386 to 814) | 1010 (798 to 1318)* | 1330 (933 to 1999)* | <0.001 |
| NTproBNP, pg/mL | 81.5 (54.8 to 98.0) | 134 (69.0 to 260)* | 184 (88.9 to 273)* | 0.002 |
| hs-TnT, ng/L | 7.2 (5.3 to 11.8) | 20.5 (10.7 to 30.2)* | 25.2 (13.4 to 34.4)* | <0.001 |

Continuous data are presented as median (interquartile range). T2D: Type II diabetes mellitus. PICP: Procollagen type I carboxy-terminal propeptide; CITP: Carboxy-terminal telopeptide of type I collagen, MMP1: Matrix Metalloproteinas 1; TIMP1: Tissue inhibitor of metalloproteinase-1; sST2: Soluble suppressor of tumorigenicity-2; MMP10: Matrix Metalloproteinas 10; NTproBNP: N-terminal brain natriuretic peptide; hs-TnT: High Sensitive Troponine. *P<0.05 versus Controls; †P<0.05 versus DM without macroalbuminuria

**Supplementary Table S2: P-values from interaction analyses assessing the association of uACR and macroalbuminuria on the associations between biomarkers and MRI parameters in T2Dpatients**

|  | **MBFwithASL** | |  | **MyocardialT1, ms** | |  | **MyocardialT2, ms** | |  | **MyocardialT2*, ms** | |
| --- | --- | --- | --- | --- | --- | --- | --- | --- | --- | --- | --- |
|  | **uACR** | **Macro**  **albuminuria** |  | **uACR** | **Macro**  **albuminuria** |  | **uACR** | **Macro**  **albuminuria** |  | **uACR** | **Macro**  **albuminuria** |
| **Biomarkers** |  |  |  |  |  |  |  |  |  |  |  |
| PICP, ng/mL | 0.672 | 0.922 |  | 0.655 | 0.794 |  | 0.544 | 0.887 |  | 0.679 | 0.815 |
| CITP, ng/mL | 0.298 | 0.529 |  | 0.360 | 0.287 |  | 0.002* | 0.021 |  | 0.468 | 0.779 |
| PICP/CITP ratio | 0.458 | 0.448 |  | 0.544 | 0.249 |  | 0.003* | 0.039 |  | 0.144 | 0.694 |
| TIMP1, ng/mL | 0.960 | 0.881 |  | 0.810 | 0.348 |  | 0.031 | 0.008 |  | 0.407 | 0.710 |
| MMP1, ng/mL | 0.632 | 0.601 |  | 0.933 | 0.905 |  | 0.832 | 0.635 |  | 0.762 | 0.744 |
| MMP1/TIMP1 ratio | 0.662 | 0.571 |  | 0.867 | 0.654 |  | 0.562 | 0.651 |  | 0.545 | 0.646 |
| sST2, ng/mL | 0.338 | 0.278 |  | <0.001*^†^ | 0.002*^†^ |  | <0.001*^†^ | 0.002*^†^ |  | <0.001*^†^ | 0.005*^†^ |
| Lipocalina, ng/mL | 0.785 | 0.200 |  | 0.953 | 0.945 |  | 0.201 | 0.125 |  | 0.724 | 0.716 |
| MMP10, pg/mL | 0.342 | 0.323 |  | 0.140 | 0.139 |  | 0.737 | 0.867 |  | 0.042 | 0.893 |
| hs-TnT, ng/L | 0.091 | 0.546 |  | 0.363 | 0.793 |  | 0.772 | 0.130 |  | 0.321 | 0.182 |
| NTproBNP, pg/mL | 0.302 | 0.430 |  | 0.435 | 0.658 |  | 0.199 | 0.007 |  | 0.786 | 0.525 |

The data are presented as P-values from interaction analyses. T2D: Type II diabetes mellitus. MBF: Myocardial Blood Flow; ASL Arterial Spin Labeling; uACR: Urine Albumin-Creatinine Ratio; CITP: Carboxy-terminal telopeptide of type I collagen; PICP: Procollagen type I carboxy-terminal propeptide; MMP1: Matrix Metalloproteinase 1; TIMP1: Tissue inhibitor of metalloproteinase-1; sST2: Soluble suppressor of tumorigenicity-2; MMP10: Matrix Metalloproteinase 10; hs-TnT: High Sensitive Troponin; NTproBNP: N-terminal brain natriuretic peptide. *P<0.05 after adjustment for multiple comparisons (FDR 5%); †P<0.05 after adjustment for age, sex and SBP. Significant P values after both adjustments are in bold.
